# Optimizing Vibratory Sorting Machine of Crickets: Effects of Surface Friction, Oscillation Dynamics, and Energy Consumption

**DOI:** 10.3390/insects17030252

**Published:** 2026-02-27

**Authors:** Arthit Duangchanchote, Sarawut Saenkham, Siripuk Suraporn, Ahmad Zainuddin, Sopa Cansee

**Affiliations:** 1 Faculty of Engineering, MahaSarakham University, Maha Sarakham 44150, Thailand; 2 Department of Biology, Faculty of Science, Mahasarakham University, Maha Sarakham 44150, Thailand; siripuk.s@msu.ac.th; 3 Faculty of Agriculture, University of Jember, Jember Regency 68121, East Java, Indonesia; zainuddin91.faperta@unej.ac.id

**Keywords:** cricket sorting, vibratory separation, static friction, surface roughness, insect farming technology, energy efficiency

## Abstract

This study presents a two-stage, mechanics-based method for optimizing vibratory sorting machine of adult crickets for post-harvest size grading. In the first stage, the static coefficient of friction (*COF*) was measured for three cricket size classes across seven tray surface conditions to quantify cricket–substrate interactions relevant to vibratory transport. *COF* varied significantly with both morphology and surface microtexture (*p* < 0.0001), with intermediate roughness levels generating higher friction than smooth or highly rough surfaces. In the second stage, a factorial experiment evaluated the effects of oscillating speed (300–350 rpm), tray inclination (2°–3°), and surface roughness (G0–G5) on sorting efficiency, throughput, batch sorting time, and specific energy consumption (*SEC*). All main factors and most interactions significantly influenced sorting performance (*p* < 0.0001). The optimal operating condition—350 rpm, 2° inclination, and G2 roughness—achieved 95% sorting accuracy, 39 crickets·min^−1^ throughput, and the lowest *SEC* (0.37 Wh·cricket^−1^). The results demonstrate that friction–vibration coupling governs cricket transport on vibrating surfaces and provide an engineering framework for designing scalable, energy-efficient sorting systems for insect rearing and processing.

## Simple Summary

Cricket farmers usually sort crickets by hand, which is slow and inconsistent. This study developed a vibratory sorting machine to automatically separate crickets by size. We first measured how crickets of different sizes interact with surfaces of varying roughness, then tested the machine under different vibration speeds, tray angles, and surface textures. The optimal combination—350 rpm, a 2° tray angle, and medium roughness—achieved 95% accuracy and high throughput while using low energy. The results show that this system can significantly reduce labor and improve efficiency in commercial cricket farming.

## 1. Introduction

Edible insect production has expanded rapidly in response to rising global protein demand, environmental constraints on conventional livestock systems, and the need for more sustainable food sources. Among farmed insects, the house cricket (*Acheta domesticus*) has emerged as one of the most commercially important species due to its high protein content, favorable amino acid profile, efficient feed conversion, and relatively low land, water, and greenhouse gas requirements [1,2,3]. From a human nutrition perspective, crickets have also been shown to be nutritionally comparable to, or in some cases healthier than, commonly consumed meats when evaluated using nutrient profiling models [4,5]. These advantages have led to the formal recognition of *A. domesticus* as a novel food ingredient by the Food and Agriculture Organization of the United Nations (FAO) [6] and to approval under the European Union Novel Food framework [7]. Beyond commercial applications, edible insects have been widely recognized as a strategic component of future food security due to their high efficiency and adaptability in resource-limited environments [8]. Thailand has become a global leader in cricket production, supported by thousands of small- and medium-scale farms supplying both domestic and international markets [9,10]. The nutritional quality and market value of crickets depend on several biological and management factors, including feed formulation [11,12,13,14], species and functional composition [15], growth stage, and rearing conditions such as temperature and humidity [16]. Sex-related differences further influence product quality, as female crickets generally exhibit higher lipid and lower protein content than males, resulting in a milder flavor preferred in certain markets [17,18]. Consequently, consistent post-harvest sorting of crickets by size—and, in some cases, by sex—is essential to meet market specifications, improve processing efficiency, and increase economic value [19,20,21].

Despite its importance, size-based sorting in most cricket farming operations remains predominantly manual. Farmers typically rely on visual inspection and hand separation, which are labor-intensive, time-consuming, and prone to interoperator variability. Manual sorting can account for a substantial share of harvesting labor, significantly increasing production costs and limiting scalability [22,23]. Advanced sorting and sexing technologies have been proposed, including high-resolution imaging combined with image processing to detect ovipositors [24,25], artificial intelligence approaches using convolutional neural networks with reported accuracies exceeding 90% [26], acoustic signal analysis of male cricket calls [27], and vision-based measurement systems employing transparent channels [28]. However, these technologies are often expensive, technically complex, and require controlled lighting, a stable power supply, and specialized expertise, thereby limiting their adoption in smallholder and decentralized farming systems [29].

In contrast, mechanical separation techniques based on vibration, oscillatory motion, and frictional differences have long been used in agricultural engineering to sort grains, seeds, and other biological materials [30]. Vibratory conveyors and vibratory sorting systems exploit interactions among gravitational forces, inertia, and surface friction to induce differential movement based on size or mass [31,32,33]. However, existing mechanical sorting systems are primarily designed for inert granular materials and are typically optimized through empirical mechanical tuning rather than by considering biological surface–object interactions. Extending these principles to cricket sorting offers a promising low-cost alternative; however, insects present a unique engineering challenge. Unlike inert granular materials, crickets possess complex tarsal structures composed of claws, adhesive pads, and secretion-mediated contact mechanisms, which strongly influence frictional behavior on solid surfaces [34,35]. Studies in insect biomechanics have shown that sliding and locomotion on inclined or vibratory substrates are governed by the balance between body weight, frictional resistance, and dynamic excitation [36,37]. Surface microtexturing plays a critical role in modulating the coefficient of friction (*COF*), with intermediate roughness often maximizing adhesion before excessive asperities reduce effective contact area [38]. Despite this knowledge, biomechanical and tribological insights have rarely been incorporated into the design or optimization of mechanical insect-sorting systems. To date, no systematic experimental study has quantified how surface roughness, oscillating speed, and inclination angle jointly influence cricket movement, sorting efficiency, and energy consumption in a vibratory system. This lack of empirical evidence represents a critical gap, hindering the rational development of affordable, efficient mechanical sorting technologies for insect farming. To address this gap, the present study developed and evaluated a vibratory sorting machine specifically designed for adult crickets. The system integrates adjustable oscillating speed, variable tray inclination, and interchangeable tray surfaces with defined micro-roughness levels. The overall structure of the study follows a two-stage experimental framework. First, the static *COF* was quantified for small, medium, and large crickets across seven surface textures to characterize frictional conditions governing sliding onset. Second, a factorial experiment examined how oscillating speed, tray inclination, and surface roughness interactively affect sorting efficiency, throughput, batch sorting time, and specific energy consumption. This stepwise structure ensures that the mechanical operating parameters are interpreted and optimized based on measured insect–surface frictional behavior rather than trial-and-error adjustment. By linking fundamental cricket surface biomechanics with full-system performance metrics, this study provides a science-based pathway for optimizing mechanical cricket sorting.

The novelty of this work lies not in proposing a new class of sorting machine, but in introducing a mechanics-informed, biotribology-guided methodology for insect sorting. Unlike prior studies that treat insect sorting as a purely mechanical or image-based classification problem, this research quantitatively links static friction behavior, surface microtexture, cricket morphology, vibratory dynamics, and energy use within a unified experimental framework. The identification of an optimal operating regime—characterized by intermediate surface roughness, shallow inclination, and high oscillating speed—demonstrates that friction–vibration coupling governs insect transport and separation. In practice, the study presents a low-cost, scalable, and energy-efficient sorting solution suitable for decentralized cricket farming systems. Scientifically, it advances understanding of friction-driven insect locomotion under vibratory excitation and provides a transferable methodology for designing automated handling and sorting technologies for other edible insect species and small biological materials.

## 2. Materials and Methods

### 2.1. Experimental Set-Up

**Figure 1:**
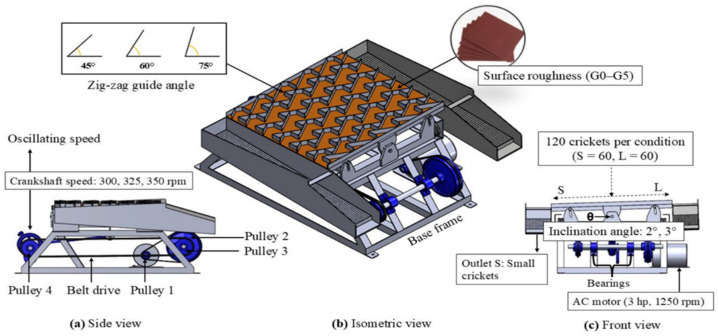
Configuration of the vibratory cricket sorting system adapted from a conventional seed sorter: (**a**) side view showing the belt–pulley and crank–shaft drive mechanism controlling oscillation speed, (**b**) isometric view of the modular sorting deck with interchangeable surface roughness plates and zigzag guide rows, and (**c**) front view illustrating the adjustable inclination angle (θ) and the two discharge outlets for small (S) and large (L) crickets.

A vibratory sorting machine, adapted from the working principles of conventional rice and seed sorting mechanisms, was used to evaluate the influence of mechanical and surface-related factors on cricket sorting performance. The system follows a standard vibratory transport architecture and was not intended as a new machine concept; rather, it was modified and configured specifically for post-harvest cricket-size sorting. The experimental platform consists of a modular oscillating sorting deck mounted on a rigid steel base frame, with adjustable inclination and interchangeable surface plates, enabling systematic investigation of operating conditions (Figure 1a–c).

The sorting deck is supported by bearings and driven by a crankshaft mechanism powered by a 3 hp AC motor. Rotational motion from the motor is transmitted through a belt–pulley system (Pulleys 1–4) to the crank assembly, which converts the rotary motion into a controlled reciprocating oscillation of the deck (Figure 1a). The oscillation speed was regulated to 300, 325, and 350 rpm by adjusting the motor input using an AC voltage controller, ensuring stable and repeatable vibration amplitudes across all tested operating conditions. Assuming one oscillation per crank revolution, these rotational speeds correspond to deck vibration frequencies of 5.00, 5.42, and 5.83 Hz, respectively, which represent the actual excitation frequencies applied to the sorting deck during the experiments. The deck inclination angle (θ) is adjustable via a screw-type mechanism, allowing the gravitational component of transport to be tuned during operation (Figure 1c). In the experiments, inclination angles ranging from 0° to 15° were used, as specified in the test matrix. The sorting tray is equipped with six parallel zigzag guide rows that impose a controlled flow path on the crickets. The guide geometry forces repeated changes in travel direction, enhancing size-dependent separation under combined vibration and gravity. Based on preliminary trials, a nominal guide turning angle of 60° was selected as it provided stable flow and improved separation efficiency. Each zigzag pathway directs crickets toward one of two discharge outlets: a small-sized outlet (S) and a large-sized outlet (L), as illustrated in Figure 1c.

To investigate the effect of frictional interactions, the deck surface is designed to be interchangeable. Surface plates are fabricated from 304 stainless steel as the baseline reference (G0) and from additional roughness grades (G1–G5), using standardized roughened sheets (Figure 1b). This modular design allows direct assessment of how surface microtexture influences cricket mobility, sliding onset, and sorting performance.

The principal structural and mechanical specifications of the machine are summarized in Table 1, including frame dimensions, motor characteristics, pulley configuration, deck geometry, and material properties. The sorting deck measures 61.5 × 70.0 × 2.5 cm (W × L × H), with each zigzag row having a width of 8 cm to accommodate the natural body size and movement patterns of adult crickets (*A. domesticus* and *Gryllus bimaculatus*). Three zigzag guide angles (45°, 60°, and 75°) are mechanically available, as listed in Table 1; however, based on preliminary trials, the 45° configuration was selected for all experiments because steeper angles (60° and 75°) promoted excessively rapid forward transport, reducing residence time on the deck and limiting effective size classification. The total deck mass of approximately 10 kg provides sufficient inertia to maintain uniform oscillatory motion under load, while the total machine weight of about 60 kg ensures mechanical stability during operation.

To ensure accurate measurement of operational parameters during each trial, the machine was equipped with multiple instruments, as listed in Table 2. A digital clamp meter measured current and voltage for energy-consumption calculations, while a laser tachometer measured oscillation speed. A precision electronic scale was used to obtain individual cricket weights during sample preparation.

### 2.2. Cricket Preparation

A mixed population of field crickets (*G. bimaculatus*) and house crickets (*A. domesticus*) was obtained from a commercial cricket farm operating under standard Thai farming practices. The use of a mixed-species population reflects common practice in smallholder and semi-industrial cricket production systems, where multiple species are often reared together due to similar husbandry requirements and the absence of species-specific market segregation [9,39]. In such systems, crickets are typically harvested and marketed as mixed batches without species differentiation [40,41]. Because the present sorting machine is designed for size-based classification rather than species discrimination, the mixed-species composition does not affect the mechanical separation process and helps ensure that the experimental conditions closely represent real post-harvest handling environments. In commercial practice, when adult crickets reach market size, they are harvested from rearing enclosures, washed and steamed, then air-dried on nylon nets to reduce surface moisture before being packed and frozen for storage and transport to buyers. The purchase price is primarily determined by size, based on buyer experience, which motivates farmers to manually sort crickets into size classes prior to sale. In this study, cricket samples were collected after air-drying and before freezing, representing a typical post-harvest, non-living state intended for commercial grading and packaging. Therefore, all specimens used in the experiments were non-living, and their motion on the sorting deck was governed purely by passive mechanical interactions (gravity, vibration, and friction), not by any biological behavior.

From the harvested batch, a 100 g sample was randomly collected. Each cricket was weighed individually using a digital balance to characterize the natural size distribution within the lot. For experimental handling and repeatability, crickets were grouped in sets of 10 individuals, and these sets were classified into three size classes based on their total group mass: small (≤4.0 g per 10 crickets), medium (4.1–7.9 g per 10 crickets), and large (≥8.0 g per 10 crickets). This grouping approach reflects practical post-harvest handling, in which crickets are commonly processed in small batches rather than individually. These group-mass thresholds correspond to approximate individual mean body masses of ≤0.40 g (small), 0.41–0.79 g (medium), and ≥0.80 g (large), as confirmed by the morphological analysis (Section 3.1, Table 3). These size groups served as the primary basis for defining the experimental factors and evaluating sorting performance.

### 2.3. Measurement of Static Coefficient of Friction

The *COF* plays a critical role in determining how crickets interact with the sorting tray during vibratory motion. Friction governs whether an insect initiates sliding, maintains stability, or becomes intermittently immobilized—behaviors that directly influence sorting accuracy and transport efficiency [34,36]. Because cricket locomotion is highly sensitive to substrate microtexture, quantifying *COF* across different surface conditions is essential for optimizing machine performance.

To evaluate frictional interactions, seven types of tray surfaces were selected. Stainless steel 304 (S304) was used as the baseline surface because of its widespread use in food-contact equipment, high corrosion resistance, and hygienic properties commonly required in insect and agricultural processing systems. In addition, six progressively roughened surfaces (G0–G5) were prepared using standardized abrasive paper (glass paper) to produce controlled increments in surface roughness. These graded surfaces were selected to provide a systematic, reproducible approximation of the continuous range of surface textures encountered in practical farm equipment, including smooth metal sheets, textured polymer trays, and roughened or worn handling surfaces. Similar approaches using graded abrasives have been widely applied in tribological studies involving biological and agricultural materials [42,43]. Thus, the G0–G5 series serves as engineering proxies for commercially available fine-to-coarse surface finishes that can be readily implemented in farm-scale sorting systems. These roughness stages enabled systematic investigation of how microtexture influences cricket–surface adhesion and sliding behavior.

Crickets from each size group (Section 2.2) were randomly selected for *COF* testing. Individual crickets were placed on the test surface attached to the machine’s adjustable inclined platform. The surface was gradually tilted from a low angle until the cricket began to slide under gravity. The critical angle at which movement began was recorded as the sliding angle. This tilt-plane method is widely recognized for its precision and suitability for measuring static friction in small biological samples [44,45]. Each combination of cricket size and surface type was tested in three replications, yielding a total of 63 friction measurements across the seven surface levels (S304 and G0–G5).

### 2.4. Vibratory Sorting Machine

The performance of the vibratory sorting machine was evaluated using 120 crickets per replication, consisting of 60 large-sized females and 60 small-sized males. These crickets were post-harvest samples obtained after steaming and air-drying, reflecting the practical processing stage used by farmers prior to freezing and sale, rather than live insects. The samples were placed onto six zigzag guiding channels, with each channel containing 10 males and 10 females. The oscillating speed, tray inclination angle, and surface roughness were set in accordance with the experimental conditions. The machine was then activated, and timing commenced simultaneously. The trial was terminated when all crickets reached their designated collection outlets or when the 10 min limit was reached. Any remaining crickets on the sorting tray after the allotted time were recorded as residual insects. Data collected per trial included sorting time and electrical energy consumption, measured from current and voltage readings. This procedure reflects realistic post-harvest handling conditions in commercial cricket production, where size-based sorting is performed on processed (non-living) crickets prior to packaging and distribution.

### 2.5. Experimental Design

**Figure 2:**
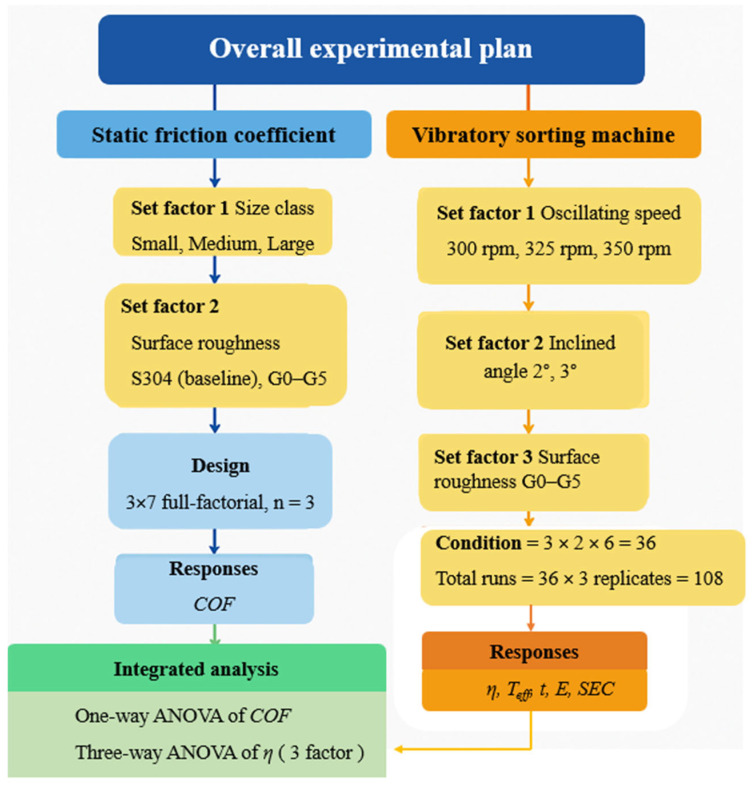
Overall experimental plan showing the two-stage testing framework: (1) *COF* measurement and (2) vibratory sorting performance evaluation.

Figure 2 illustrates the overall experimental framework, which was structured into two sequential components: first, to characterize the fundamental frictional behavior of post-harvest crickets on engineered surfaces, and second, to evaluate the operational performance of the vibratory sorting machine. Conducting the study in two steps ensured that the mechanical parameters applied in the sorting trials were supported by empirical friction data, thereby improving the validity and interpretability of the sorting performance results.

#### 2.5.1. Static Coefficient of Friction Test

The first phase of the experimental design quantified how cricket size and tray-surface microtexture influence the onset of sliding—an essential determinant of cricket mobility on vibratory platforms. Friction governs the interaction between insects and supporting substrates, shaping their stability, adhesion, and propensity to slip under mechanical disturbances [34,36]. Because vibratory sorting relies on controlled passive sliding, accurate characterization of the *COF* is fundamental to optimizing tray surface design and overall sorting performance. Two experimental factors were examined: (i) cricket size (small, medium, and large) and (ii) tray surface material (stainless steel S304 and six graded roughness levels, G0–G5). Stainless steel S304 was selected as the baseline surface because it is a widely used food-grade material in insect and agricultural processing environments due to its corrosion resistance, smoothness, and hygienic properties [46]. The additional six roughness grades (G0–G5) were prepared using standardized abrasive surfaces to emulate progressively increasing surface microtexture. Such controlled roughness gradients are commonly applied in tribological studies to evaluate biological adhesion and sliding responses [42,43].

The factorial combination of size class and surface type produced 21 treatment conditions, each replicated three times, yielding a total of 63 *COF* measurements. These results provided mechanistic insight into how substrate microtexture either facilitates or inhibits cricket sliding. The quantitative friction profiles obtained in this step were subsequently used to interpret the observed movement behavior during mechanical sorting and to inform parameter selection for the second phase of the experiment.

#### 2.5.2. Vibratory Sorting

Following the friction assessment, the second phase of the study evaluated how key operational parameters of the vibratory sorting machine influence sorting performance. The selected parameters were based on established principles of vibratory conveying, in which oscillation frequency, amplitude, and inclination angle collectively determine transport velocity, trajectory, and material stratification behavior [31,47]. Because the cricket sorter relies on differential mobility governed by friction and inertia, these mechanical factors are expected to strongly influence sorting outcomes. This experiment incorporated three operational factors: sorting plate speed (300, 325, and 350 rpm), tray inclination angle (2° and 3°), and surface roughness (G0–G5), yielding 36 treatment combinations. With three replications per combination, a total of 108 sorting trials were conducted. The collected data were analyzed in terms of sorting efficiency (*η*), defined as the proportion of correctly classified crickets; effective throughput, defined as the number of correctly sorted crickets per minute; batch sorting time, defined as the total time required for each run; and *SEC*, defined as the energy required per correctly sorted cricket.

By integrating controlled-friction data from Step 1 with sorting-performance outcomes from Step 2, the study determined not only which operational settings yielded superior performance but also why those settings were effective. This two-stage experimental framework therefore provides a robust mechanistic basis for interpreting sorting behavior and for identifying optimal configurations suitable for commercial-scale cricket sorting systems.

### 2.6. Performance Metrics

To evaluate the performance of the vibratory cricket sorting machine, several metrics were defined and calculated using the following equations.

#### 2.6.1. Static Coefficient of Friction (*COF*)

(1) $$\mu_{s}=\tan(\alpha c)$$

where *αc* is the critical tilt angle (°) at which the cricket begins to slide on the test tray. $\mu_{s}$ is dimensionless. The angle *αc* was obtained using the inclined-plane (tilt) method with quasi-static tilting; see, e.g., Ibrahim [42], Bachchhav & Bagchi [43], Pajic-Lijakovic et al. [44], and Mohsenin [45].

#### 2.6.2. Sorting Efficiency (*η*)

(2) $$\eta \;(\%)=\frac{N_{correct}}{N_{total}}\;\times \;100$$

where $N_{correct}$ is the number of correctly sorted crickets (sum of the diagonal of the confusion matrix), and $N_{total}$ = 120 is the total number of crickets loaded per run. This metric is standard in classification/separation studies [48,49,50].

#### 2.6.3. Effective Throughput ($T_{eff}$)

(3) $$T_{\mathrm{eff}}=\frac{N_{correct}}{t}$$

where *t* is the batch sorting time per run (min) measured from start to completion of the fixed batch, using $N_{correct}.$

#### 2.6.4. Electrical Energy Consumption (*E*)

*E* = *V* × *I* × *th* (4)

where *V* and *I* are the RMS voltage (*V*) and current (*A*) averaged over the run, and *th* = *t*/60 is operating time in hours.

#### 2.6.5. Specific Energy Consumption (*SEC*)

(5) $$\mathrm{SEC}=\frac{E}{N_{correct}}$$

where *E* is total energy consumed per run (Wh), $N_{correct}$ is the number of correctly sorted crickets.

### 2.7. Statistical Analysis

Statistical analyses were performed using IBM SPSS Statistics 29. The General Linear Model (GLM) was used to evaluate the significance of the experimental factors. Analysis of variance (ANOVA) was conducted in two forms: one-way ANOVA was used to assess differences in *COF* across cricket size groups and tray surface materials, while three-way ANOVA was applied to evaluate the effects of oscillating speed, inclination angle, and surface roughness on sorting performance. A significance level of *p* < 0.05 was adopted for all tests. In addition, correlation analyses were conducted to examine the relationships between *COF* and the sorting machine’s key response variables, including *η*, *Teff*, and *SEC*. These analyses were used to identify the operating conditions that yielded optimal performance.

## 3. Results

### 3.1. Morphological Characteristics and Size Distribution of Crickets

Adult crickets used in the experiments were categorized into three size classes based on the group-mass criteria defined in Section 2.2 (i.e., total mass of 10 crickets per group). When expressed on an individual basis, these classes corresponded to the following body mass ranges: small (≤0.40 g), medium (0.41–0.79 g), and large (≥0.80 g). Clear morphological gradients were observed across the three size classes (Table 3). The mean individual body mass increased from 0.30 ± 0.07 g in the small group to 0.64 ± 0.07 g in the medium group and 0.98 ± 0.10 g in the large group. Similarly, both body length and body width increased systematically with size class, confirming consistent scaling of external morphology with body mass.

The size-class distribution in the 100 g batch (Table 4) showed that medium-sized crickets dominated the sample (80 individuals, 47.21% of total mass), followed by large (42 individuals, 38.27%) and small crickets (34 individuals, 11.42%). This distribution reflects typical post-harvest conditions, in which crickets are collected at market size, resulting in a population skewed toward medium- and large-sized individuals.

### 3.2. Static Coefficient of Friction Across Surface Roughness Grades

The *COF* differed significantly across surface roughness grades and cricket size classes (Table 5). One-way ANOVA revealed highly significant effects of surface type for all size groups (*p* < 0.0001), with coefficients of determination (*R*^2^) of 0.958 for small, 0.976 for medium, and 0.921 for large crickets, indicating that surface texture accounted for most of the variance in the friction measurements.

**Figure 3:**
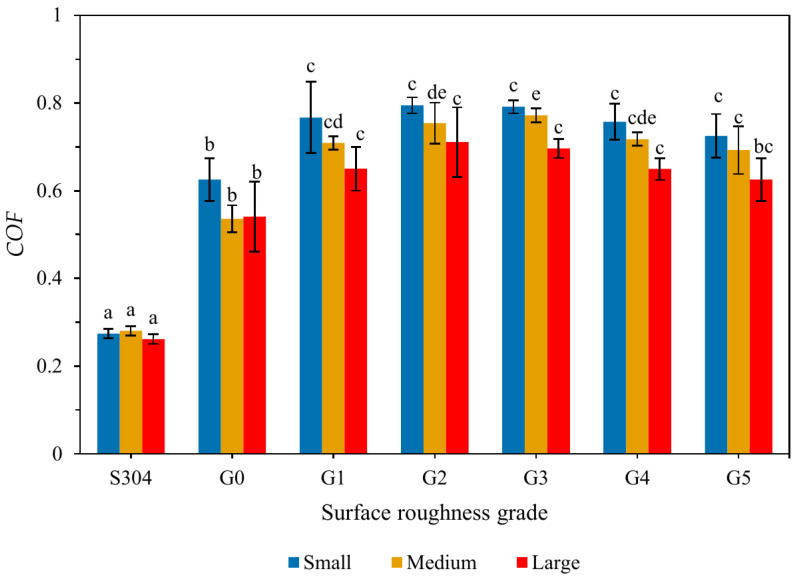
Static friction coefficient (*COF*) of crickets on different surface roughness grades (S304, G0–G5) for three size classes. Bars show mean ± SD. Different letters indicate significant differences (ANOVA with Tukey’s HSD test, *p* < 0.05); bars sharing the same letter are not significantly different.

Across all size classes, the baseline stainless-steel surface (S304) and the smoothest roughness level (G0) produced the lowest *COF* values, whereas intermediate roughness grades (G1–G3) generated substantially higher friction (Figure 3).

At the highest roughness levels (G4–G5), *COF* values tended to plateau or decrease slightly. Medium-sized crickets consistently exhibited higher *COF* values than small or large crickets across most surface conditions, indicating a size-dependent frictional response. These trends demonstrate that surface roughness and cricket size jointly influence frictional behavior and sliding resistance on the sorting tray. The G0–G5 roughness range provided sufficient contrast in sliding behavior to support the subsequent machine performance tests presented in Section 3.3.

### 3.3. Effects of Operating Parameters on Sorting Performance

A three-way ANOVA revealed that oscillating speed, inclination angle, and surface roughness each exerted a highly significant influence on sorting efficiency (*p* < 0.0001 for all main effects), with the overall model accounting for 97.8% of the variance in the response (*R*^2^ = 0.978; Table 6). Significant two-way interactions were observed between oscillating speed × surface roughness and inclination angle × surface roughness (*p* < 0.0001), indicating that the effect of mechanical excitation and gravity on sorting performance depends strongly on the frictional properties of the tray surface. In addition, the three-way interaction among oscillating speed, inclination angle, and surface roughness was significant (*p* < 0.0001), indicating that optimal sorting performance arises from the combined influence of these three parameters.

In contrast, the interaction between oscillating speed and inclination angle alone was not statistically significant (*p* = 0.0773). This indicates that, within the tested range, these two parameters primarily act additively rather than synergistically. In practical terms, changes in oscillating speed produced similar trends in sorting efficiency at both inclination angles, and changes in inclination angle produced consistent effects across the tested speed levels, unless surface roughness was also varied.

#### 3.3.1. Oscillating Speed and Inclination Angle

**Figure 4:**
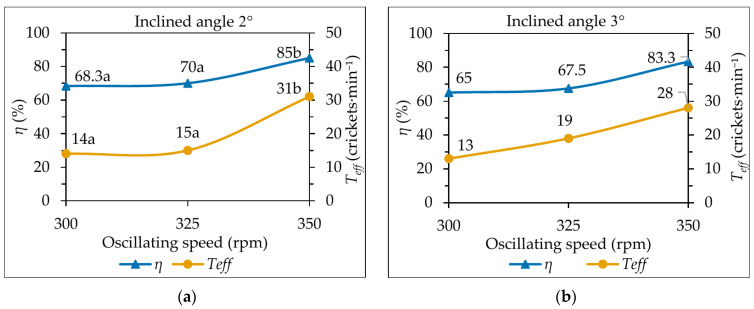
Effect of oscillating speed on sorting efficiency (*η*) and effective throughput ($T_{eff}$) at (**a**) 2° and (**b**) 3° inclination angles. Different letters indicate significant differences among oscillating speeds within each inclination angle (ANOVA with Tukey’s HSD test, *p* < 0.05).

At a fixed inclination angle of 2°, increasing the oscillating speed from 300 to 350 rpm increased sorting efficiency from 68.3% to 85.0%, while effective throughput rose from 14 to 31 correctly sorted crickets·min^−1^ (Figure 4a). At an inclination angle of 3°, sorting efficiency increased from 65.0% to 83.3% and throughput from 13 to 28 crickets·min^−1^ over the same speed range (Figure 4b). These results show that higher oscillating speeds consistently enhanced both sorting accuracy and processing capacity. However, for a given speed, the higher inclination angle (3°) generally resulted in slightly lower effective throughput than 2°, indicating that steeper slopes reduce the residence time of crickets on the deck and thus decrease the opportunity for size-based separation.

#### 3.3.2. Surface Roughness

**Figure 5:**
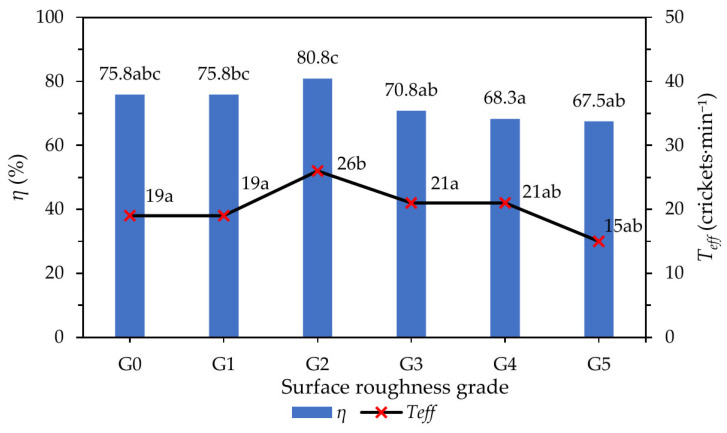
Effect of surface roughness on sorting efficiency (*η*) and effective throughput ($T_{eff}$). Different letters indicate significant differences among surface roughness grades (ANOVA with Tukey’s HSD test, *p* < 0.05).

Surface roughness had a strong influence on both sorting efficiency and throughput. Among the six roughness grades (G0–G5) evaluated, the intermediate roughness level G2 produced the highest sorting efficiency (approximately 80.8%) and the highest effective throughput (approximately 26 crickets·min^−1^). In contrast, both the smoothest surface (G0) and the roughest surface (G5) resulted in lower efficiencies (approximately 68–76%) and reduced throughputs (approximately 15–21 crickets·min^−1^) (Figure 5).

This non-linear response indicates that neither minimal nor excessive surface roughness is optimal for sorting. Moderate roughness provides sufficient resistance to regulate sliding and prevent premature loss of smaller crickets, whereas excessively rough surfaces disrupt smooth transport, leading to unstable motion, intermittent blockages, or reduced flow continuity.

### 3.4. Energy Consumption and Optimal Operating Conditions

**Figure 6:**
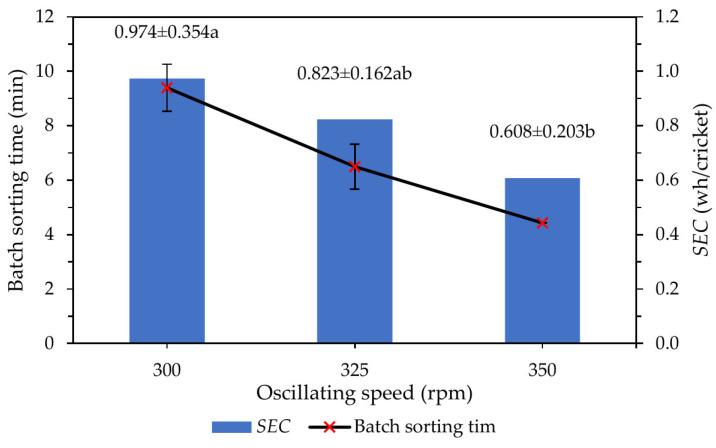
Effect of oscillating speed on specific energy consumption (*SEC*) and batch sorting time. Different letters indicate significant differences among oscillating speeds (ANOVA with Tukey’s HSD test, *p* < 0.05).

Sorting time and energy consumption were strongly influenced by the selected operating parameters, particularly oscillating speed and inclination angle (Figure 6). Under the optimized surface condition (G2), increasing the oscillating speed and reducing the inclination angle led to faster processing and lower *SEC* (Table 7). At an inclination angle of 2°, the system achieved a batch sorting time of 3.1 min, an effective throughput of 39 crickets·min^−1^, and a sorting efficiency of 95%. The corresponding energy consumption per run was 42.4 Wh, yielding an *SEC* of 0.37 Wh·cricket^−1^. In contrast, at the same oscillating speed and surface roughness (G2) but with a steeper inclination angle of 3°, batch sorting time increased to 4.13 min, effective throughput decreased to 29 crickets·min^−1^, and energy consumption per run increased to 56.5 Wh, resulting in a higher *SEC* of 0.50 Wh·cricket^−1^.

Although sorting efficiency remained constant at 95% for both inclination angles, the shallower slope (2°) clearly improved processing capacity and reduced energy demand per sorted cricket. These results indicate that energy efficiency in the vibratory sorting system is governed not only by sorting accuracy, but also by the combined effects of residnce time, transport velocity, and friction-controlled motion on the deck surface.

## 4. Discussion

This study investigated the combined effects of cricket morphological variation, surface microtextured, and vibratory operating parameters on the performance of a newly developed mechanical sorting system. By integrating frictional characterization with machine-level performance testing, the results provide mechanistic insight into how biological and engineering factors interact to determine sorting accuracy, throughput, and energy efficiency. The findings have practical implications for post-harvest handling in commercial cricket farming and contribute to an emerging body of research on insect biomechanics and processing technologies.

### 4.1. Morphology-Dependent Friction and Sorting Efficiency

Cricket size and body morphology exerted a significant influence on sliding behavior and frictional resistance on the sorting surfaces. In particular, medium-sized crickets consistently exhibited higher static coefficients of friction across the tested surface types, indicating stronger effective contact with the substrate and greater resistance to sliding. This trend is consistent with the measured morphometric differences among size classes (Section 3.1), in which body mass, width, and length increased systematically from small to large individuals, as previously reported for *A. domesticus* and *G. bimaculatus* [9,10].

From a biomechanical perspective, size-dependent differences in contact area, tarsal pad geometry, and limb force distribution are known to influence insect adhesion and locomotor stability. Prior studies have demonstrated that insect attachment performance and gait mechanics scale with body mass and limb morphology, thereby affecting frictional interactions and slip resistance on solid substrates [34]. The higher *COF* values observed for medium-sized crickets in the present study can, therefore, be interpreted as a consequence of an optimal balance between body mass and effective contact area, which enhances substrate adhesion under static and vibratory conditions.

The predominance of medium- and large-sized individuals in the sampled batch reflects typical harvesting practices in commercial cricket farming, where insects are collected close to marketable size [39]. This population structure has direct implications for machine performance, as different size classes exhibit distinct slip thresholds and transport responses under vibratory excitation. Consequently, overall sorting efficiency is governed not only by mechanical parameters such as oscillation speed, inclination, and surface roughness, but also by intrinsic biological variability in morphology and frictional behavior.

These findings highlight that the design and optimization of automated cricket-sorting systems must explicitly account for morphology-dependent frictional interactions, rather than relying solely on kinematic or purely mechanical considerations. Incorporating such biotribological effects provides a more robust basis for predicting insect motion on engineered surfaces and for achieving reliable size-based separation in practical post-harvest processing systems.

### 4.2. Surface Microtexture–Dependent Friction and Cricket–Substrate Interaction

Surface roughness exerted a strong influence on cricket mobility, producing a clear non-linear frictional response across the tested substrates. Intermediate roughness levels (G1–G3) consistently yielded the highest *COF* values, promoting controlled sliding and reducing erratic or unstable motion during transport. This behavior is consistent with tribological studies showing that moderate microtexture enhances mechanical interlocking and adhesive interactions of biological attachment structures, whereas surfaces that are either too smooth or too rough reduce the effective contact area and traction efficiency [35,43].

At the highest roughness grades (G4–G5), the slight decline or plateau in *COF* suggests that excessive asperities disrupt pad conformability and fluid-mediated contact mechanisms, thereby limiting frictional force generation [34,36]. Similar trends have been reported in studies of insect locomotion and substrate interaction, where optimal adhesion occurs at intermediate roughness scales rather than at extreme surface textures [37].

These results demonstrate that effective surface engineering for mechanical insect sorting should focus on optimizing microtexture rather than simply increasing roughness. The frictional characterization presented in Section 2.5.1, therefore, provides a critical mechanistic basis for interpreting the sorting performance outcomes discussed in Section 2.5.2 and for identifying surface conditions that enable predictable, stable sliding dynamics in vibratory sorting systems.

### 4.3. Mechanical Parameters Governing Sorting Efficiency Through Vibration–Friction Interactions

Oscillating speed, inclination angle, and surface roughness jointly governed cricket sorting behavior, as evidenced by the significant main effects and interaction terms observed in the statistical analysis. Increasing oscillating speed enhanced transport velocity and improved separation efficiency, consistent with established principles of vibratory conveying and particulate transport dynamics [31,47]. However, these performance gains were strongly modulated by surface roughness, demonstrating that vibratory excitation alone is insufficient to ensure efficient sorting unless frictional conditions support stable, controlled sliding. The inclination angle played a subtler but still critical role in regulating sorting performance. A shallow slope (2°) increased the residence time of crickets on the sorting deck, thereby enhancing the probability of size-dependent separation. In contrast, a steeper inclination (3°) shortened contact duration with the surface, promoting premature sliding and reducing effective throughput. This behavior reflects the need to balance gravitational driving forces against frictional resistance to maintain controlled transport and separation, as reported in previous studies on insect and small-particle handling under vibratory conditions [51,52].

Taken together, these results demonstrate that optimal sorting performance does not arise from adjusting any single mechanical parameter in isolation. Instead, efficient and reliable separation arises from the coupled interactions among vibratory excitation, gravitational forcing, and surface friction, which act on biological bodies with size-dependent mechanical responses. This interaction-based perspective underscores the importance of system-level optimization over parameter-by-parameter tuning in the design of mechanical sorting technologies for edible insects.

### 4.4. Energy Use and System Optimization for Sustainable Insect Processing

The energy analysis demonstrates that mechanical optimization of vibratory sorting systems can significantly improve both processing efficiency and energy performance without compromising sorting accuracy. Although sorting efficiency remained constant at 95% under the optimized surface condition (G2), substantial differences in batch time, throughput, and *SEC* across inclination angles indicate that energy demand is governed primarily by transport dynamics rather than by classification accuracy alone.

At the lower inclination angle (2°), the increased residence time on the deck allowed crickets to undergo controlled, friction-mediated transport and size-based separation while maintaining a high forward flow rate. This operating regime reduced overall processing time and minimized wasted mechanical work associated with premature sliding or unstable motion. In contrast, the steeper angle (3°) reduced the contact time between crickets and the surface, resulting in lower throughput and higher energy consumption per run, although sorting efficiency remained unchanged. Similar trade-offs between transport speed, residence time, and energy efficiency have been reported in studies of vibratory conveyors and particulate transport systems [31,47].

These results highlight that energy-efficient sorting cannot be achieved simply by increasing vibration intensity or slope. Instead, optimal performance arises from tuning the coupled interactions among vibration, gravity, and surface friction, which collectively govern transport stability and flow continuity. This interaction-based control of energy use is consistent with previous findings in vibratory handling and separation systems, where excessive driving forces often increase energy demand without proportional gains in productivity [47,53]. From an applied perspective, the identified operating regime—high oscillating speed, moderate surface roughness (G2), and shallow inclination—provides a practical guideline for designing low-energy, high-throughput sorting systems for post-harvest insect processing. Such mechanical optimization is particularly relevant for decentralized and small- to medium-scale cricket farms, where energy costs and equipment simplicity are critical constraints [39,54]. Moreover, improving the energy efficiency of post-harvest handling contributes directly to the broader sustainability goals of edible insect production, which is increasingly promoted as a low-impact protein source compared with conventional livestock systems [12,55].

Overall, these findings demonstrate that mechanically simple, friction-informed vibratory systems can deliver both high productivity and low energy consumption, offering a scalable and cost-effective alternative to labor-intensive manual sorting or high-cost sensor-based technologies in commercial cricket processing chains.

### 4.5. Practical and Scientific Implications of the Two-Stage Framework

The combined approach—first quantifying frictional behavior and then evaluating machine performance—provides a robust mechanistic basis for explaining and predicting cricket-sorting outcomes. The strong correspondence between *COF* patterns and sorting results confirms that surface–insect interactions are central to vibratory sorting systems. This dual-phase design offers a methodological template for future research on automated insect processing or mechanical handling of other small biological organisms. From an industry perspective, the optimized sorting system substantially increases throughput while reducing labor input, addressing a key bottleneck in cricket farming operations. It also offers a pathway toward standardized size grading, improving market quality and processing efficiency. Scientifically, the study advances understanding of how biological variation, surface engineering, and vibratory mechanics interact, thereby contributing to a growing field linking biomechanics with agri-automation and insect technology.

From a practical implementation perspective, maintenance requirements are an important consideration for adoption in small- and medium-scale cricket farming systems. The proposed vibratory sorting system relies on a mechanically simple architecture consisting primarily of a motor, belt–pulley transmission, crank mechanism, bearings, and interchangeable surface plates. These components are standard industrial parts with well-established maintenance procedures and readily available replacements. Routine maintenance would primarily involve periodic inspection and lubrication of bearings, belt tension adjustment or replacement, and cleaning of the sorting deck to maintain hygienic conditions for food-contact surfaces. The roughened surface plates (G0–G5), which are subject to gradual wear due to repeated frictional contact, are designed to be modular and easily replaceable, allowing surface performance to be restored at low cost without modifying the core machine structure. Importantly, no complex sensors, electronics, or calibration-intensive subsystems are required, thereby reducing technical maintenance demands and improving operational robustness under field conditions. This simplicity supports the system’s suitability for decentralized production environments, where ease of servicing, low downtime, and minimal technical expertise are critical for long-term use.

## 5. Conclusions

This study demonstrates that the performance of a vibratory sorting machine for crickets is governed by the coupled effects of cricket morphology, surface microtexture, and vibratory operating parameters. By integrating friction-based characterization of cricket–substrate interactions with factorial performance testing of a mechanically adapted sorting system, this work establishes a mechanistic and application-oriented framework for optimizing size-based separation in post-harvest insect processing. The static coefficient of friction varied strongly with both surface roughness and cricket size, confirming that locomotion stability and slip thresholds are inherently morphology-dependent. Intermediate microtextured surfaces (G1–G3) consistently exhibited higher friction, whereas excessively smooth or overly rough substrates reduced effective adhesion and the predictability of movement. These frictional trends were directly reflected in sorting performance. An operating regime combining moderate roughness (G2), shallow inclination (2°), and high oscillating speed (350 rpm) delivered the best overall performance, achieving 95% sorting accuracy, a throughput of 39 crickets·min^−1^, and the lowest specific energy consumption (0.37 Wh·cricket^−1^). These results confirm that sorting efficiency cannot be optimized through mechanical settings alone but rather arises from the coordinated interaction between biological traits and engineered surface conditions.

Beyond the specific system studied, the two-stage experimental framework provides broader methodological value. Frictional characterization proved to be a powerful predictive tool for interpreting machine behavior, highlighting the central role of surface engineering in insect-handling technologies. From an industrial perspective, the optimized configuration offers a low-cost, scalable, and energy-efficient solution that can substantially reduce labor requirements and improve grading consistency in cricket production systems—an increasingly important need as insect farming continues to industrialize.

Scientifically, this work contributes to the fields of biotribology, vibratory transport, and automated insect processing by clarifying how adhesion mechanics, surface microtexture, and vibration jointly govern the movement of small biological organisms. Overall, the proposed framework supports the development of efficient, robust, and scalable sorting technologies for edible insects and can be readily adapted to other insect species and mechanical post-harvest processing applications, thereby supporting the continued growth of sustainable insect farming systems.

## Figures and Tables

**Table 1:** Mechanical and structural parameters of the vibratory cricket sorting machine.

Parameter	Value/Unit
**Base frame unit**	
Base frame dimensions	63.5 × 70.0 × 30.0 cm
Motor specification	3 hp, 1250 rpm
Frame material	Mild steel
Crank shaft	13.5 cm
Pulley 1 (motor)	5.1 cm
Pulley 2 (main shaft)	20.3 cm
Pulley 3 (crankshaft)	10.2 cm
Pulley 4 (camshaft)	10.2 cm
Power system	Belt drive
**Parameter**	**Value/Unit**
**Base frame unit**	
Slope angle at outlet	4°
Cricket outlet dimension	15.0 × 93.0 cm
Machine weight	60 kg
**Sorting deck unit**	
Deck dimensions	61.5 × 70 × 2.5 cm
Zig-zag guide angle	45°, 60°, 75° (45° used in experiments)
Width of each row	8 cm
Number of zig-zag rows	6 rows
Surface roughness levels	Stainless steel 304; Ra grades G0–G5
Inclination angle	0–15° adjustable
Deck vibration speed	300, 325, 350 rpm
Deck vibration frequency	5.00, 5.42, 5.83 Hz
Deck weight	10 kg

**Table 2:** Instrumentation used for measuring electrical, mechanical, and mass-related variables during experimentation.

Instrument	Measured Parameter	Model	Range	Accuracy
AC voltage controller	Motor input voltage	Fasizi AC 220 V	0–100% of 220 V AC	±1%
Digital Clamp Meter	AC A; AC/DC V	KEW SNAP200	A: 0–400 A; V: 0–600 V	±2%
Electronic Scale	Mass	KA67/K1918B	0–50 g	±0.01 g
Laser tachometer	Oscillation speed	DT-2234C	1–9999 rpm	±0.05%

**Table 3:** Morphological characteristics of crickets in different size classes (mean ± SD).

Size Class	Individual Body Mass Range (g)	Mean Body Mass (g)	Body Width (mm)	Body Length (mm)
Small	≤0.40	0.3 ± 0.07	6.67 ± 0.41	19.45 ± 0.68
Medium	0.41–0.79	0.64 ± 0.07	7.77 ± 0.55	23.90 ± 0.52
Large	≥0.80	0.98 ± 0.10	8.65 ± 0.75	27.46 ± 1.35

**Table 4:** Size-class distribution and mass contribution of crickets in the 100 g sampled batch.

Size Class	Number of Crickets (*n*)	Total Mass of Group (g)	Proportion of Sample (%)
Small	34	11.42 ± 3.01	11.42
Medium	80	47.21 ± 5.80	47.21
Large	42	38.27 ± 6.21	38.27

**Table 5:** One-way ANOVA of the static friction coefficient (*μ_s_*) across surface roughness grades within each size class.

Size Class	*R* ^2^	SS	df	MS	*F* Value	Sig.
Small cricket	0.958	0.627	6	0.104	52.841	*p* < 0.0001
Medium cricket	0.976	0.552	6	0.092	92.938	*p* < 0.0001
Large cricket	0.921	0.434	6	0.072	27.109	*p* < 0.0001

**Table 6:** ANOVA for the effect of operational parameters on sorting efficiency.

Source	SS	df	*F* Value	Sig.
Oscillating speed	6718.806	2	804.719	*p* < 0.0001
Inclined angle	199.811	1	47.863	*p* < 0.0001
Surface roughness	2310.002	5	110.669	*p* < 0.0001
Oscillating speed × Inclined angle	22.158	2	2.654	0.0773
Oscillating speed × Surface roughness	1423.454	10	34.098	*p* < 0.0001
Inclined angle × Surface roughness	1886.277	5	90.369	*p* < 0.0001
Oscillating speed × Inclined angle × Surface roughness	1003.069	10	24.028	*p* < 0.0001
Error	300.573	72		
Total	590,959.410	108		

Note: *R*^2^ = 0.978. Significance tested at α = 0.05.

**Table 7:** Performance of the vibratory cricket sorting machine at optimized parameters.

Parameter	Oscillating Speed (rpm) × Surface Roughness G2	
	Inclined Angle 2°	Inclined Angle 3°
Batch sorting time (min)	3.1	4.13
Effective throughput (crickets/min)	39	29
Sorting efficiency (%)	95	95
Energy per run (Wh)	42.4	56.5
*SEC* (Wh/cricket)	0.37	0.50

## Data Availability

The original contributions presented in this study are included in the article. Further inquiries can be directed to the corresponding author.
